# Infographic. The first position statement of the Concussion in Para Sport Group

**DOI:** 10.1136/bjsports-2021-104530

**Published:** 2021-10-05

**Authors:** Richard Weiler, Cheri Blauwet, David Clarke, Kristine Dalton, Wayne Derman, Kristina Fagher, Vincent Gouttebarge, James Kissick, Kenneth Lee, Jan Lexell, Peter Van de Vliet, Evert Verhagen, Nick Webborn, Adam Virgile, Osman Hassan Ahmed

**Affiliations:** 1 Amsterdam Collaboration on Health & Safety in Sports, Department of Public and Occupational Health, Amsterdam Movement Sciences, Amsterdam UMC, University Medical Centers – Vrije Universiteit Amsterdam, Amsterdam, The Netherlands; 2 Sport & Exercise Medicine, Fortius Clinic, London, UK; 3 Para Football Foundation, Arnhem, The Netherlands; 4 Department of Physical Medicine and Rehabilitation, Spaulding Rehabilitation, Spaulding Hospital/Brigham and Women’s Hospital, Harvard Medical School, Boston, Massachusetts, USA; 5 Kelley Adaptive Sports Research Institute, Boston, Massachusetts, USA; 6 University of Hertfordshire, Hatfield, Hertfordshire, UK; 7 School of Optometry & Vision Science, University of Waterloo, Waterloo, Ontario, Canada; 8 Institute of Sport and Exercise Medicine, Division Orthopaedic Surgery, Dept of Surgical Sciences, Faculty of Medicine and Health Sciences, Stellenbosch University, Cape Town, South Africa; 9 IOC Research Center, Cape Town, South Africa; 10 Rehabilitation Medicine Research Group, Department of Health Sciences, Lund University, Lund, Sweden; 11 Amsterdam UMC, University of Amsterdam, Department of Orthopaedic Surgery, Amsterdam Movement Sciences, Meibergdreef 9, Amsterdam, The Netherlands; 12 Section of Sports Medicine, University of Pretoria, Pretoria, South Africa; 13 Department of Family Medicine, University of Ottawa, Ottawa, Ontario, Canada; 14 Medical Committee, International Paralympic Committee, Bonn, Nordrhein-Westfalen, Germany; 15 Spinal Cord Injury/Disorder, Physical Medicine & Rehabilitation, Medical College of Wisconsin, Milwaukee, Wisconsin, USA; 16 Immune-Oncological Centre Cologne, Cologne, Germany; 17 Former Medical & Scientific Director International Paralympic Committee, Bonn, Nordrhein-Westfalen, Germany; 18 Department of Public and Occupational Health, EMGO, Amsterdam UMC Locatie VUmc, Amsterdam, Netherlands; 19 Centre for Sport and Exercise Science and Medicine, University of Brighton, Eastbourne, UK; 20 School of Sport, Exercise and Health Sciences, Loughborough University, Loughborough, UK; 21 College of Nursing and Health Sciences, University of Vermont, Burlington, Vermont, USA; 22 Physiotherapy Department, University Hospitals Dorset NHS Foundation Trust, Poole, UK; 23 University of Portsmouth School of Sport Health and Exercise Science, Portsmouth, Hampshire, UK

**Keywords:** disabled persons, sport, brain concussion, head, position statement

## Background

A concussion is a common injury in many sports, including para sport. Aside from a more comprehensive need for concussion education, clinicians face difficulties applying concussion assessment and management guidelines to para athletes.^1^ At present, there is a lack of para-sport concussion research, and prior International Concussion in Sport (CIS) consensus papers have not addressed this specific population. To rectify this issue and improve concussion management provided to para athletes, the Concussion in Para Sport (CIPS) multidisciplinary expert group was created.^2^

## Methods

The CIPS group undertook an in-depth analysis of issues specific to the para athlete within the established key clinical domains of the current (2017) Consensus Statement on Concussion in Sport.^3^ The existing Sports Concussion Assessment Tool 5 (SCAT5) was evaluated as part of this process and helped identify para athlete-specific concerns. Four CIPS working groups were tasked with exploring the following key clinical areas of concussion in para sport described in the most recent consensus statement of concussion in sport^2^:

- Concussion assessment;
- Concussion management;
- Return-to-sport following concussion; and
- Specific considerations related to the different impairments in para athletes.

## Recommendations

Regular preparticipation and periodic health examinations in the para athlete are essential to determine a baseline reference point for concussion symptoms but pose challenges for the interpreting clinician.

Concussion in the para athlete population should be managed according to existing concussion consensus guidelines using the CIPS assessment tools (see https://bjsm.bmj.com/content/bjsports/suppl/2021/04/09/bjsports-2020-103696.DC1/bjsports-2020-103696supp001_data_supplement.pdf, https://bjsm.bmj.com/content/bjsports/suppl/2021/04/09/bjsports-2020-103696.DC1/bjsports-2020-103696supp002_data_supplement.pdf). Paradoxically, while SCAT5 baseline testing cannot be mandated for para athletes, the clinician attending to a para athlete with a suspected concussion has a much greater need to have a comprehensive understanding of a para athlete’s preinjury cognitive function and physical abilities to make a diagnosis of concussion and manage the athlete more effectively.

Due to the lack of validity of the SCAT5 in general populations and even greater variability of baseline scores between different disability groups,^4^ a para athlete may have a concussion even if his or her SCAT5 is deemed to be ‘normal’. Despite their limitations, regular preparticipation and periodic health examinations along with the SCAT5 help guide the assessment of a suspected concussion for each para athlete impairment group.

As part of the overall assessment, an attending medical professional may choose to seek a corroborative history from suitable family members, caregivers or members of the athlete’s entourage who are familiar with the athlete’s baseline level of function, if available, to assist in clinical decision-making. In addition, it is strongly recommended that a team clinician with prior knowledge of the athlete is involved in the acute assessment of the potentially concussed athlete. Further considerations for concussion management of the para athlete are required within the remove, rest, reconsider and refer consensus statement framework. Considering a return to sport (RTS), the 2017 CIS consensus statement has limitations when considering the RTS of the para athlete. Case-by-case decision-making related to RTS following concussion is imperative.

## Future directions

Additional challenges exist for the evaluation and management of concussion in para athletes. Looking ahead, further research is needed to develop a greater understanding of existing knowledge gaps and attitudes towards concussion among athlete medical staff, coaches and para athletes themselves. Future research should investigate the use, reliability and validity of common assessment tools in the para athlete population. Concussion prevention strategies and sport-specific rule changes, such as in Para Alpine Skiing,^5^ Cerebral Palsy Football^6^ and sports for athletes with visual impairment, should also be considered to reduce the occurrence of concussion in para athletes.

**Figure:**
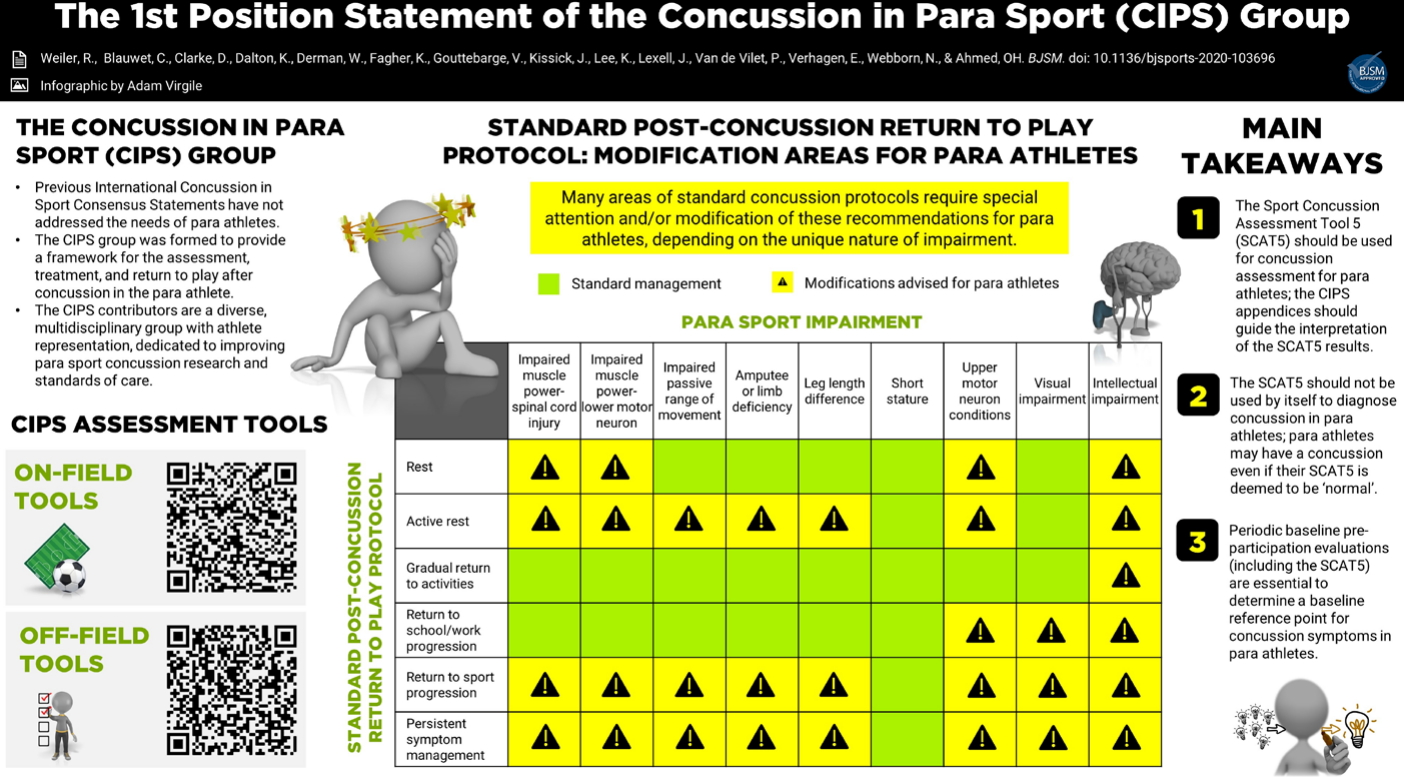


## References

[1] Derman W , Runciman P , Schwellnus M , et al . High precompetition injury rate dominates the injury profile at the Rio 2016 Summer Paralympic Games: a prospective cohort study of 51 198 athlete days. Br J Sports Med 2018;52:24–31. 10.1136/bjsports-2017-098039 29030389

[2] Weiler R , Blauwet C , Clarke D , et al . Concussion in para sport: the first position statement of the concussion in para sport (CIPS) group. Br J Sports Med 2021. 10.1136/bjsports-2020-103696. [Epub ahead of print: 09 Apr 2021]. PMC8551975 33837003

[3] McCrory P , Meeuwisse W , Dvořák J , et al . Consensus statement on concussion in sport-the 5^th^ international conference on concussion in sport held in Berlin, October 2016. Br J Sports Med 2017;51:838–47. 10.1136/bjsports-2017-097699 28446457

[4] Weiler R , van Mechelen W , Fuller C , et al . Do neurocognitive SCAT3 baseline test scores differ between footballers (soccer) living with and without disability? A cross-sectional study. Clin J Sport Med 2018;28:43–50. 10.1097/JSM.0000000000000407 28107219

[5] Blauwet C , Webborn N , Kissick J , et al . When van Mechelen's sequence of injury prevention model requires pragmatic and accelerated action: the case of para alpine skiing in Pyeong Chang 2018. Br J Sports Med 2019;53:1390–1. 10.1136/bjsports-2018-099997 30967380

[6] Ahmed OH , Fulcher M , Malone D . The introduction of temporary concussion substitutions in disability football: Are we ‘headed’ in the right direction? Football Medicine & Performance 2020;32:13–17.

